# Decreased Plasma COMP and Increased Plasma CTX-II Levels in a Chinese Pseudoachondroplasia Family with Novel* COMP* Mutation

**DOI:** 10.1155/2017/5809787

**Published:** 2017-08-27

**Authors:** Chongjuan Gu, Zhao Yang, Hao Tan, Yingying Zhang, Yilu Lu, Yongxin Ma

**Affiliations:** Department of Medical Genetics, West China Hospital, Sichuan University, Chengdu, China

## Abstract

Pseudoachondroplasia (PSACH) is an autosomal dominant osteochondrodysplasia caused by mutations in the gene encoding cartilage oligomeric matrix protein (COMP). Accurate clinical diagnosis of PSACH is sometimes difficult. Here, we identified a novel* COMP* mutation (c.1675G>A, p.Glu559Lys) in a Chinese PSACH family. We detected the plasma levels of COMP and type II collagen (CTX-II) in the four affected individuals. The results showed the levels of plasma COMP significantly decreased and plasma CTX-II significantly increased in the three PSACH patients with COMP mutation. However, both plasma levels of COMP and CTX-II were not to have found significant difference between the presymptomatic carrier and the age-matched subjects.* In vitro* analysis and immunofluorescence displayed wild type COMP homogenously expressed in cytoplasm, but mutant proteins were irregularly accumulated inside the HEK-293 cells. Western blot revealed that the quantity of the mutant COMP was more compared to wild type COMP in cells after transfection for 12 hours and 24 hours. Subsequently, 3D structural analysis showed three changes have taken place in secondary structure of the mutant COMP. In conclusion, the novel mutation of* COMP* may result in intracellular accumulation of the mutant protein. Decreased plasma COMP and increased plasma CTX-II may potentially serve as diagnostic markers of PSACH but may not be applicable in the presymptomatic carrier.

## 1. Introduction

Pseudoachondroplasia (PSACH; MIM# 177170) is an autosomal dominant osteochondrodysplasia characterized by disproportionate short stature, brachydactyly, joint laxity, scoliosis, early onset osteoarthritis, and epiphyseal and metaphyseal abnormalities. The affected individual is normal at birth and usually recognized at 2-3 years of age on the basis of a waddling gait and decreased linear growth [1, 2]. Joint pain is the most debilitating feature of PSACH beginning in childhood and persisting throughout life. Radiographic findings commonly include abnormalities of the tubular bones, with metaphyseal and epiphyseal irregularity, scoliosis, lumbar lordosis, and anterior beaking of the vertebral bodies [3]. But PSACH patients generally have normal craniofacial appearance and intelligence.

PSACH is believed to be caused exclusively by mutations in the gene encoding cartilage oligomeric matrix protein (COMP), which located on chromosome 19p13.1 [4–6]. COMP, also known as thrombospondin 5 (TSP-5), is an extracellular matrix (ECM) protein and is mainly expressed in cartilage and bone tissue [7, 8]. COMP is a pentameric protein and each monomer is comprised of four domains: an N-terminal pentamerization domain, an epidermal growth factor-like domain, a type 3 (calcium-binding) repeat domain (type 3 repeat), and a C-terminal globular domain (CTD) [9, 10]. Mutation in* COMP* also results in multiple epiphyseal dysplasia (MED; MIM# 132400), a relatively milder skeletal dysplasia and genetically heterogeneous disorder. To date, more than 100 mutations in* COMP* have been reported in about 300 patients with either PSACH or MED, but without any mutations in exons 15, 17, and 19 of* COMP* having been identified [11, 12].

Diagnosis of PSACH is usually based primarily on family history, clinical manifestation, physical examination, radiographic evaluation, and genetic diagnosis. However, accurate diagnosis of PSACH sometimes may be difficult, especially for nonexpert doctors, and because clinical presentation varies substantially between PSACH individuals, most radiographic changes are not disease-specific and DNA testing is expensive and not used widely [13]. There is evidence that reduced plasma COMP levels may serve as a diagnostic marker in adult PSACH patients [14, 15]. But circulating COMP levels in children younger than 3 years of age with COPM mutations have not been studied, and those children carry COMP mutations but have not yet showed symptoms of PSACH. Besides, COMP, as a crucial ECM protein, is known to interact with other ECM components such as collagens [16]. Type II collagen (CTX-II) can be detected in serum and its level has been shown to correlate with Kashin-Beck disease or osteoarthritis [17]. However, circulating CTX-II levels in individuals with COMP mutations and potential utility as a biomarker have not been explored.

In this study, we identified a novel variant (c.1675G>A, p.Glu559Lys) in exon 15 of* COMP* in a Chinese PSACH family with four affected members in three generations. The plasma levels of COMP and CTX-II in the affected individuals were determined.* In vitro* expressions of normal and mutant COMP protein were performed to investigate the supposed pathological mechanism. Additionally, to explore the influence of the variant reported in this study on the protein structure and function, three-dimensional homology models for the normal and mutant proteins were built.

## 2. Materials and Methods

### 2.1. Subjects and Skeletal Radiograph Analysis

**Figure 1:**
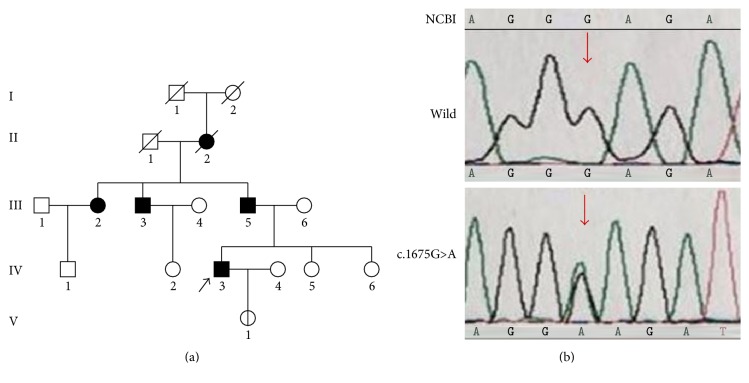
Family pedigree and sequencing results of affected individuals and normal members. (a) Family pedigree: the proband was indicated by arrow and the presymptomatic carrier was indicated by the unfilled, single vertical lines symbol. (b) Sequencing results: the site of the variant was marked by arrows.

**Figure 2:**
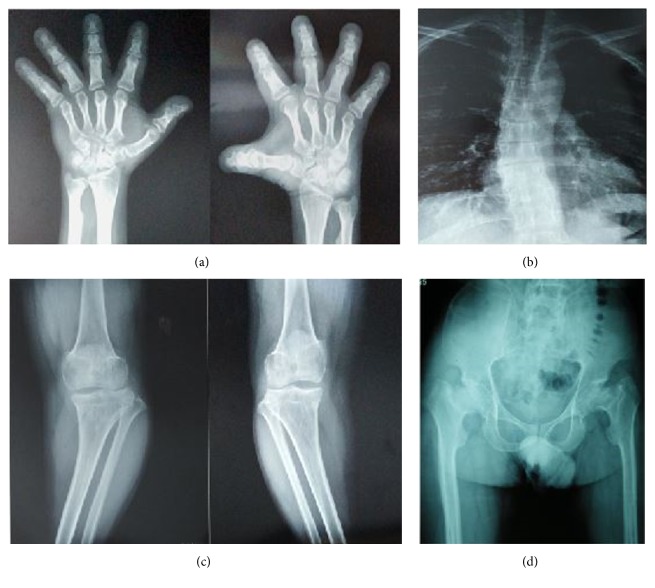
Radiological skeletal survey of the proband. (a) The hands were small. Phalanges and metacarpals were short and thick with cone-shaped epiphyses. (b) There was scoliosis and widening of the interpedicular distances from upper to lower spine. (c) The tibiae and fibulae were mildly shortened and knee varus. (d) Pelvis radiogram showed increased acetabular angles and narrowed hip joint space and flattened and irregular femoral heads with shortened necks.

The subjects are the three dwarf members (individuals III-3, III-5, and IV-3) and the child (individuals V-1) of a Han nationality family in Southwest China (see pedigree in Figure 1(a)). The grandma of the proband (individual IV-3) was the first dwarf individual who died at age of 76. She has had one daughter and two sons, and all were affected with dwarf (height 138–145 cm), osteoarthrosis, and scoliosis. The proband, 34-year-old and 140 cm in height, is the only affected child of individual III-5 whose two sisters are normal. He is an intelligent man with normal craniofacial appearance, short stature, disproportionately short limbs, and short fingers. He suffered knee and hip joint pain during adolescence followed by severe osteoarthritis. Skeletal radiograph showed short metacarpals and phalanges, tibial plate dysplasia, scoliosis, and flattened and irregular femoral heads with shortened necks, increased acetabular angles, and narrowed hip joint space (Figure 2). He underwent bilateral hip replacement surgery to relieve pain and improve function. His only daughter, 2.5 years old, is 88 cm height and in normal gait.

According to the family history and clinical characteristics of the proband, PSACH was suggestive diagnosis and* COMP* mutation was analyzed for the definitive diagnosis. This study was approved by ethics committees of West China Hospital, Sichuan University, and informed consent was obtained from the patients and family members.

### 2.2. *COMP* Mutation Detection and Validation

Firstly, genomic DNA (gDNA) was extracted from blood samples of the proband using standard method (QIAGEN, Germany). Exons 8–19 and their flanking intronic sequence of the COMP were amplified using eight pairs of primers (Supplement Table  1, in Supplementary Material available online at https://doi.org/10.1155/2017/5809787) by polymerase chain reaction (PCR) and subjected to DNA sequencing. A heterozygous nucleotide change c.1675G>A (p.Glu559Lys) was identified in exon 15 (nucleotide numbering is according to cDNA sequence with GenBank accession number XM_009336). Secondly, we amplified and sequenced the* COMP* exon 15 in the two affected individuals: III-3 and III-5, as well as the daughter of proband (V-1). Thirdly, PCR and sequencing of the* COMP* exon 15 in three unaffected individuals: IV-2, IV-5, and IV-6 were performed. Lastly, PCR and sequencing of the candidate variant were performed on gDNA from 100 unaffected individuals of the same ethnic background as the patients. Two different software programs including PolyPhen-2 (http://genetics.bwh.harvard.edu/pph2) and SIFT (http://sift.jcvi.org/) were used to predict whether the amino acid substitution affects protein function.

### 2.3. Plasma COMP and CTX-II Assay

Four individuals with the* COMP* variant (c.1675G>A) of the family participated this study, and they were individuals III-3, III-5, IV-3, and V-1. The control group were selected at random (i.e., in terms of any given health condition) from the West China Hospital, Sichuan University, and participants selected were, respectively, matched to the age of the four affected individuals. Three milliliters of peripheral blood was collected into an EDTA-containing tube for plasma separation. The blood samples were centrifuged and the plasma was aspirated and stored at −80°C until further processing. Plasma COMP and CTX-II concentrations of the four affected individuals and twelve controls were analyzed using enzyme linked immunosorbent assay kit (USCN Business Co., Wuhan, China) according to the manufactures' protocol. Each measurement was performed in triplicate.

### 2.4. Construction of Plasmids

Total RNA was extracted from human leukocytes of peripheral blood using TRIzol reagent (QIAGEN, Germany) and RNA was reverse transcribed into cDNA (Thermo Fisher Scientific Inc., USA). PcDNA3.1 expression vectors of human wild type and mutant type (c.1675G>A)* COMP* was constructed following the standard procedure of the Ligation-Free-Cloning Kit (ABM, Canada). Thereinto, DNA insert fragments were amplified using special primer (Supplement Table  2) including the homologous sequence of vector and desired gene-specific region. In the meanwhile, the linearized pcDNA3.1 vector carrying Myc tag was prepared using restriction enzyme digest method. Afterwards, cloning procedure was set up as standard protocol, and selected colonies were confirmed through PCR amplification following gel electrophoresis and plasmid extraction following sequencing.

### 2.5. Transient Transfection and Western Blot

Approximately 1 × 10^5^ HEK-293 cells were plated 24 hours before transfection. Cells were transiently transfected with 2 ug of each pcDNA3.1-wtCOMP or pcDNA3.1-mtCOMP or pcDNA3.1-myc empty plasmid using jetPRIME® transfection (Polyplus-transfection SA, France) according to the manufacturer's protocol. HEK-293 cells were incubated with the transfection reagents for 4 h at 37°C with 5% CO_2_ in DMEM supplemented with 1% antibiotics and 10% fetal calf serum, followed by further 12 hours or 24 hours of incubation in fresh full DMEM. After incubation, cells were lysed in lysis buffer followed by determination of protein concentration with BCA Protein Assay Kit (Beyotime Institute of Biotechnology, Beijing, China).

Prepared proteins were subjected to Western blot for myc, and GADPH was used as an internal control. In brief, proteins were subjected to SDS-PAGE, transferred to PVDF membrane, and detected with specific appropriate primary antibodies and horseradish peroxidase-conjugated secondary antibodies. Specific proteins were visualized using an enhanced chemiluminescence (ECL) Western blot detection system. Semiquantification of mean band intensities by Quantity One was shown.

### 2.6. Immunofluorescence

The immunofluorescence was performed using a standard protocol. The anti-Myc mouse antibody (Santa Cruz Biotechnology Inc., CA, USA) was used as primary antibody in combination with a rabbit anti-Grp78 antibody (Zen BioScience, Chengdu, China), a representative marker of endoplasmic reticuluma, each in a 1 : 100 dilution. As secondary antibodies the anti-mouse-DyLight 594-labeled antibody and the anti-rabbit-DyLight 488-labeled antibody (ZSGB-Bio, Beijing, China) were used in 1 : 100 dilutions, respectively. The cells were mounted with DAPI (Sigma, USA) for nuclear staining and the images were acquired with laser scanning confocal microscope (Olympus, Japan).

### 2.7. Structural Analysis

To explore the effect of the novel variant on the protein structure and function, 3D models for the normal and mutant proteins were analyzed. Wild type COMP was downloaded from PDB (http://www.rcsb.org/pdb/explore/explore.do?structureId=3fby); ID: 3FBY is the crystal structure of the signature domain of COMP including amino acid sequence of 225–757 with resolution of 3.15 Å [18]. The mutant (p.Glu559Lys) COMP was built using SWISS MODEL (https://www.swissmodel.expasy.org/) based on the 3FBY for template [19, 20]. At the end of modeling, the global and per-residue model quality was assessed using the QMEAN scoring function. The structural representations were generated with PyMOL, a Python based viewer for visualization of macromolecular structures.

### 2.8. Statistics

Statistical analyses were performed using SPSS Version 17.0 software (SPSS Inc., Chicago, IL, US). All data were presented as the mean ± standard deviation using GraphPad Prism 5.0 software. Differences between groups were determined using the nonparametric Mann–Whitney* U *test and Student's* t*-test. *P* values of less than 0.05 were considered statistically significant.

## 3. Results

### 3.1. *COMP* Mutation Analysis

Three PSACH patients in this family (individuals III-3, III-5, and IV-3) were heterozygous for the nucleotide change c.1675G>A (Figure 1(b)) in exon 15, which is located at the CTD of COMP. This sequence change was predicted to result in a substitution of amino acid 559 from glutamic to lysine in COMP (p.Glu559Lys). The proband's daughter (V-1) also was detected to have the same heterozygous variant, namely, presymptomatic carrier. The novel variant was not identified in three unaffected family members (IV-2, IV-5, and IV-6) and the 100 unaffected individuals. The p.Glu559Lys mutation was predicted to be “probably damaging” with scores of 1.0 by the PolyPhen-2 and was predicted as “deleterious” with scores of −3.693 by the SIFT.

### 3.2. Plasma Concentrations of COMP and CTX-II

**Figure 3:**
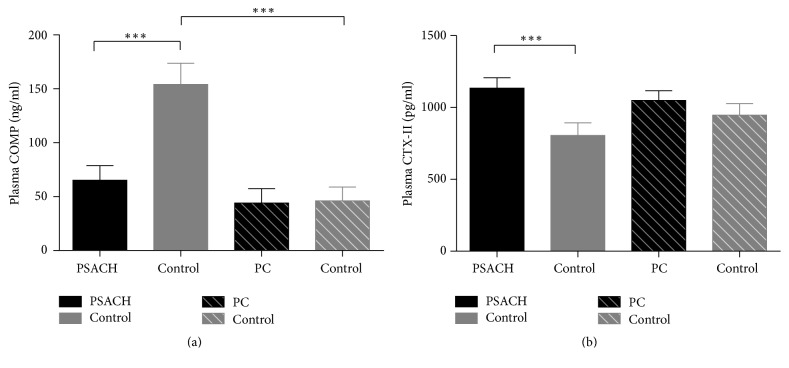
Plasma levels of COMP and CTX-II in the affected individuals and age-matched subjects. (a) Plasma COMP levels in PSACH were significantly lower than the age-matched controls, and plasma COMP levels in normal adult controls were significantly higher than the normal children less than three years old. (b) Plasma CTX-II levels in PSACH were significantly increased compared with the age-matched controls; ^*∗∗∗*^*P* < 0.001.

As shown in Table 1, the mean plasma COMP concentrations of three PSACH patients with COMP mutation was 65.33 ± 13.37 ng/ml, which was significantly lower than the concentrations of age-matched controls, 154.14 ± 19.69 ng/ml (*P* < 0.001). But, in the proband's daughter (presymptomatic carrier), the mean plasma COMP level had no difference with equal-aged controls (*P* = 0.864). Besides, the mean plasma COMP level was significantly higher in the adult controls than in the controls less than three years old (*P* < 0.001) (Figure 3(a)). As for CTX-II, the mean plasma concentrations of three PSACH patients with COMP mutation were 1134.28 ± 72.19 pg/ml, which was significantly higher than the concentrations of age-matched controls, 805.04 ± 88.00 pg/ml (*P* < 0.001) (Table 1). But, in the presymptomatic carrier, the plasma CTX-II level had no significant differences with equal-aged subjects (*P* = 0.497). Also, there was no discrepancy of CTX-II levels in normal adults and children (*P* = 0.887) (Figure 3(b)).

### 3.3. Characteristics of Wild Type and Mutant COMP Expressed in HEK-293 Cells

**Figure 4:**
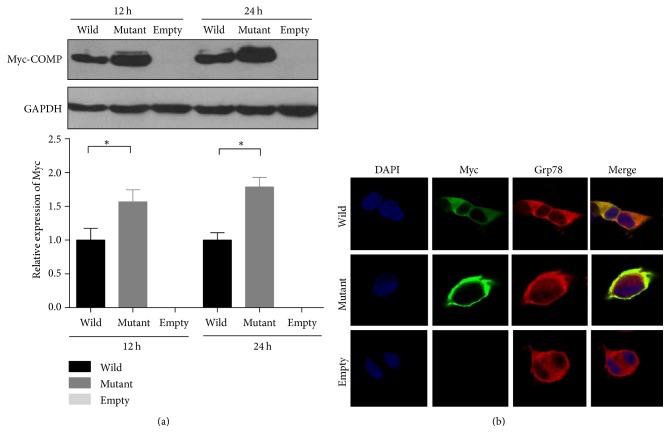
Comparison of processes of secretion of wild type and mutant COMP. HEK-293 cells were transfected with expressed plasmids of wild type COMP, mutant COMP, and empty vector, respectively, and for immunofluorescence and Western blot analysis. (a) Western blot after transfection of 12 hours and 24 hours showed the levels of mutant COMP in cells were more than wild type COMP. (b) Immunofluorescence after transfection of 24 hours showed that wild type COMP homogenously expressed in cytoplasm and mutant protein irregularly accumulated inside the cell membrane; ^*∗*^*P* < 0.05.

The results of Western blot detecting the transfected cells using anti-myc showed mutant and wild type proteins were expressed in HEK-293 cells. And, also, quantity of the mutant COMP in cell was significantly more than wild type COMP after transfection for 12 hours and this trend was more pronounced after 24 hours (Figure 4(a)). Meanwhile, cell immunofluorescence showed that the wild type COMP was homogenously expressed in cytoplasm after transfection for 24 hours, but, interestingly, mutant proteins were irregularly accumulated inside the cell membrane (Figure 4(b)).

### 3.4. Structural Characterization

**Figure 5:**
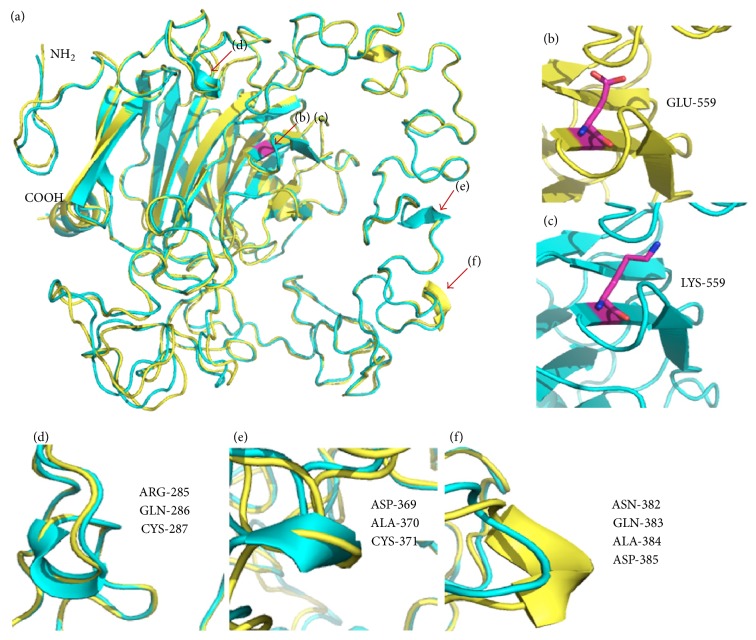
Comparison of the wild type and mutant COMP proteins. (a) Structural superimposition of the wild type COMP (yellow) and mutant COMP (cyan): purple in the *β*-strand showed the mutation site and it is amplified in (b) wild type and (c) mutant type. The changed secondary structures were amplified in (d), (e), and (f), respectively.

The crystal structure used in this study was COMP protein that includes the last epidermal growth factor repeat, the type 3 repeats, and the CTD [18]. The CTD is a *β*-sandwich that is composed of 15 antiparallel *β*-strands, and the novel mutation is located on *β*3 strand (Figure 5(a)), a substitution of amino acid 559 from glutamic to lysine (Figures 5(b) and 5(c)). To properly visualize the crystal structure differences between the native and mutant proteins, we spatially superimposed the molecules (Figure 5(a)). The results showed that three changes have taken place in secondary structure of mutant COMP protein, and those, respectively, were loops changed to helixes in amino acids 285–287 and amino acids 369–371 and helix to loop in amino acids 382–385 (Figures 5(d), 5(e), and 5(f)).

## 4. Discussion

In this study, we report a PSACH family with six affected members in Southwest China. The five PSACH patients were all in dwarf with disproportionately short limbs and short fingers and severe osteoarthrosis and scoliosis. Through* COMP* mutation detection, we identified a novel variant (c.1675G>A, p.Glu559Lys) in exons 15 of* COMP* in the three PSACH patients and an asymptomatic carrier. The variant was not detected in the three unaffected individuals in this family and 100 individuals of the same ethnic background, which demonstrate this variant is pathogenic mutation and not a single nucleotide polymorphism. This is the first report of the variant in exons 15 (aa 557–572) of* COMP* in PSACH and/or MED patients.

There is evidence that peripheral blood COMP levels decrease in PSACH patients with COMP mutations, but circulating COMP levels in asymptomatic carrier have not been reported [14, 15]. Our results further confirmed the decreased plasma COMP in Chinese PSACH patients. Furthermore, our results also illustrated that the plasma COMP concentration in normal children less than 3 years old was obviously lower than in adults and was normal before the* COMP* mutation carrier showing the clinical symptom of PSACH. The mechanism for the lower circulating COMP in children remains unclear, probably because the children under three years of age do not begin rapidly epiphyseal development, and the chondrocytes express relatively fewer COMP. This also interprets why affected individuals do not exhibit the symptom of PSACH before 2-3 years of age.

As a crucial ECM protein, COMP binds to type II and type I collagens in cartilage and tendons, respectively, for providing the tissues with resistance to tensile forces. And COMP interacts mainly with free type I and type II collagen molecules for promoting further assembly [16]. Since plasma COMP levels in PSACH patients were decreased, we hypothesized CTX-II levels in those PSACH patients would increase, thus exploring another potential biomarker. Out results demonstrated that increased plasma CTX-II concentration may potentially be used as diagnostic marker of PSACH patients. Similarly, the plasma CTX-II level in the presymptomatic carrier was not differed from those age-matched normal children. Those results are consistent with the plasma levels of COMP; as a pair of interacting proteins, when extracellular COMP decrease, the free CTX-II may correspondingly remain and increase.

Subsequently, we performed in vitro expression of normal and mutant COMP protein to investigate the supposed mechanism. Early electron microscopy studies of PSACH iliac crest biopsies showed dilated rough endoplasmic reticulum (rER) in chondrocyte [21]. Subsequent studies displayed that mutant COMP is misfolded and accumulated in the rER resulting in excessive ER stress and ultimately premature chondrocyte death [10, 22]. The study of transgenic mouse and in vitro experiments all observed intracellular trafficking of mutant COMP [23, 24]. In this study, similar results suggest the wild type COMP may be normally secreted into the extracellular matrix, but the mutant protein is intracellular accumulation. Besides, we observed more cells death in mutant COMP than wild type in 48 hours after transfection (data not shown). We supposed that the mutant COMP may be retained in the chondrocyte and cause increasing rER stress and cells death and then result in chondrocyte dysplasia and osteoarthritis.

Approximately 90% of the reported COMP mutations were identified in the type 3 repeat but only 9% in CTD [12]. CTD of COMP is highly conserved region between all TSPs, which means this structure is important for the function of the COMP. And most of the mutations mapped to the CTD were reported to impact the interaction of CTD with the type 3 repeats. Disrupt inter- or intramolecular interactions and the proper folding of the type 3 repeats are proposed to be the effects of the mutations in CTD [25, 26]. In addition, the type 3 repeats and the CTD have a net charge as negative at pH 7, and mutations in CTD also have significant effect on the local charge balance and overall change distribution [18]. The novel mutation in this study and the acidic glutamic acid residues with negative charge in 559 changed to basic lysine residues with positive charge, and there were three changes in secondary structure of type-3 repeats in the 3D models of structural superimposition. The two changes from loop to helix occurred in disulfide bond (C282–C287 and C351–371) of type 3 repeats, and the change from helix to loop was in the potential metal-ion-dependent adhesion site of type 3 repeats, which are all essential for maintaining protein function [18]. Therefore, we suppose that mutation of Glu559Lys takes place through disrupting the local charge balance to change the secondary structure of type 3 repeats and thus impacts the correct folding of the protein.

In summary, our findings expand the spectrum of mutations in COMP leading to PSACH. We illustrate that the novel mutation (Glu559Lys) changes the secondary structure of COMP, which possibly leads to misfolding and intracellular retention of mutant COMP proteins. Importantly, we suggest decreased plasma COMP and increased plasma CTX-II may serve as a pair of diagnostic markers of PSACH patient but may not be applicable in the presymptomatic carrier. However, there are still some limitations in our paper. Except for intracellular pathogenic mechanisms, it has been reported that mutations in CTD of COMP could allow secretion causing PSACH by interference of the collagen fiber assembly and disruption of ECM structure in cartilage and tendons [27, 28]. Further study should be performed to demonstrate if the extracellular mechanism contributes to the COMP mutation (Glu559Lys) causing PSACH. Besides, we could not elucidate how the novel mutation changed the distant secondary structure in 3D model. Crystal structure analysis needs to confirm this change and thermodynamic and kinetic studies are required for exploring the exact mechanisms.

## Supplementary Material

Supplement table 1: List of primers used for amplification of exons 8–19 of the* COMP* gene.
Supplement table 2: List of primers used for amplification of cloning insert fragment.

## Figures and Tables

**Table 1:** Plasma levels of COMP and CTX-II in the affected individuals and age-matched controls.

Group	*n*	Age range	COMP (ng/ml)		CTX-II (pg/ml)	
			Mean ± SD	*P*	Mean ± SD	*P*
PSACH	3	35–65	65.33 ± 13.37	0.000	1134.28 ± 72.19	0.000
Control	9	34–64	154.14 ± 19.69		805.04 ± 88.00	
PC	1	2.5	44.05 ± 13.37	0.864	1049.84 ± 67.29	0.497
Control	3	2-3	46.05 ± 12.89		947.13 ± 79.25	

Plasma levels of COMP were significantly higher in the adult control than in the controls less than three years of age, *P* < 0.001. Plasma levels of CTX-II were not different between the adult controls and the controls less than three years of age, *P* = 0.887. PC: presymptomatic carrier.
