# Supplementary material for: Decreased Plasma COMP and Increased Plasma CTX-II Levels in a Chinese Pseudoachondroplasia Family with Novel COMP Mutation
Source: Biomed Res Int. 2017 Aug 27;2017:5809787. doi: 10.1155/2017/5809787 (PMC5591969; doi:10.1155/2017/5809787)
Supplement: Supplementary file 1 — Supplement table 1: List of primers used for amplification of exons 8–19 of the COMP gene. Supplement table 2: List of primers used for amplification of cloning insert fragment. [file 5809787.f1.docx]

Supplement table 1 List of primers used for amplification of exons 8–19 of the *COMP* gene

| primer | Sequence 5′→3′ | Product size (bp) |
| --- | --- | --- |
| 8F | GCTTGAGGCGGGGTTGGGTG | 433 |
| 9R | ACCGTGCCGAGCCGTAGATC |  |
| 10F | ACGGAGTGTGACCTTTGCCTTCCT | 384 |
| 10R | CATAGTCCAGCTTACCCCATCCT |  |
| 11F | GCTAAGTCATTCTGGCCTGGTC | 518 |
| 12R | AGCGTTTTGTCAAAGGCTACCGA |  |
| 13F | CGGGTAGCCTTTGACAAAACG | 391 |
| 13R | GCCCGCCCACCGTAGACAC |  |
| 14F | GGCGGGCCCTGACTTTAGCA | 548 |
| 15R | ATAACCCCGCCCCTCTGTTG |  |
| 16F | GTTCTGGGTGCCAGGTTCAT | 375 |
| 16R | AAGGGTTTTACGGAGGGTCAT |  |
| 17F | TGCTCCCAACTGTCTCTCCATG | 412 |
| 17R | ACCTGGGCCTGTGTGTCCATC |  |
| 18F | TCTGAGAGGGAAGGGTCTGG | 443 |
| 19R | CCCTTCTCACTTCCCCCTCA |  |

Supplement table 2 List of primers used for amplification of cloning insert fragment

| Pragment | Primer | Sequence 5′→3′ |
| --- | --- | --- |
| wt COPM | S | ACCGAGCTCGGATCCATGGTCCCCGACACCGCCTGCGTTCTT |
|  | AS | AACGGGCCCTCTAGACTAGGCTTGCCGCAGCTG |
| mt COMP | 1- S | ACCGAGCTCGGATCCATGGTCCCCGACACCGCCTGCGTTCTT |
|  | 1-AS | CTGTTCATTGTCTGCACGATCTTCCTTCCCTGGTT |
|  | 2- S | GGAAGATCGTGCAGACAATG |
|  | 2-AS | AACGGGCCCTCTAGACTAGGCTTGCCGCAGCTG |
